# Development of Embodied Word Meanings: Sensorimotor Effects in Children’s Lexical Processing

**DOI:** 10.3389/fpsyg.2016.00317

**Published:** 2016-03-08

**Authors:** Michelle Inkster, Michele Wellsby, Ellen Lloyd, Penny M. Pexman

**Affiliations:** Language Processing Laboratory, Department of Psychology, University of CalgaryCalgary, AB, Canada

**Keywords:** sensorimotor, auditory naming, imageability, body–object interaction, language development, semantic processing

## Abstract

Previous research showed an effect of words’ rated body–object interaction (BOI) in children’s visual word naming performance, but only in children 8 years of age or older (Wellsby and Pexman, 2014a). In that study, however, BOI was established using adult ratings. Here we collected ratings from a group of parents for children’s BOI experience (child-BOI). We examined effects of words’ child-BOI and also words’ imageability on children’s responses in an auditory word naming task, which is suited to the lexical processing skills of younger children. We tested a group of 54 children aged 6–7 years and a comparison group of 25 adults. Results showed significant effects of both imageability and child-BOI on children’s auditory naming latencies. These results provide evidence that children younger than 8 years of age have richer semantic representations for high imageability and high child-BOI words, consistent with an embodied account of word meaning.

## Introduction

Theories of embodied cognition emphasize the importance of sensorimotor experience for acquiring and representing conceptual knowledge (e.g., Glenberg, 2015). The embodied cognition framework has had a strong influence in recent research on adult language processing. It has received less research attention in the developmental literature and yet, as Wellsby and Pexman (2014b) argued, in order to refine theories of embodied cognition it is important to consider and fully integrate developmental findings. The purpose of the present study was to investigate whether children’s previous sensorimotor experience with words’ referents influences their lexical processing of those words.

From an embodied cognition perspective, representations of word meaning are, at least in part, grounded in sensorimotor and other bodily systems (for a review see Meteyard et al., 2012). Hence, it should be possible to observe effects of sensorimotor information on lexical processing. Indeed, there is now extensive research examining the effects of imageability in visual word recognition tasks (e.g., Strain et al., 1995; Cortese et al., 1997). Imageability is characterized as a word’s ability to arouse mental imagery (visual, sound, etc., Cortese and Fugett, 2004). Typically, word recognition is facilitated for words with referents that can be more easily imaged/sensed.

While imageability primarily measures *sensory* experience with words’ referents, Siakaluk et al. (2008a) have characterized a measure that is more focused on *motor* experience with words’ referents. This is the body–object interaction variable (BOI) and ratings of this dimension capture how easily a human body can interact with a word’s referent. Research with adults shows that in lexical decision tasks, phonological lexical decision tasks, and semantic categorization tasks, words rated high in BOI (e.g., belt) are processed faster and/or more accurately than words rated low in BOI (e.g., ship; Siakaluk et al., 2008a,b; Tillotson et al., 2008). To be clear, these are all concrete, highly imageable words, and they differ only in rated ease of bodily interaction; word frequency, length, and other factors are matched across high BOI and low BOI word sets. The proposed explanation is that previous motor experience with words’ referents (as measured by the BOI variable) facilitates word recognition.

Siakaluk and colleagues have explained BOI effects in adult word recognition in terms of feedback activation in the lexical system. That is, in a fully interactive word recognition system comprised of orthographic, phonological, and semantic units (e.g., Harm and Seidenberg, 2004), it is assumed that words with richer semantic representations provide stronger feedback to the orthographic and phonological units. As a result, orthographic and phonological units are faster to settle into stable patterns of activation for words with relatively richer semantic representations. In most word recognition tasks, activity in the orthographic and phonological units provides the basis for responding (e.g., Hino and Lupker, 1996). If sensorimotor information is part of lexical semantic knowledge, then high BOI words will have richer semantic representations than low BOI words, and phonological and orthographic processing will be facilitated via semantic feedback. Similarly, high imageability words are presumed to have richer semantic representations than low imageability words and thus imageability effects emerge in lexical processing tasks.

While we now understand a great deal about how sensorimotor effects like BOI and imageability influence adult lexical processing, we know much less about when they begin to influence lexical processing in children. In the first study to explore development of BOI effects in children, Wellsby and Pexman (2014a) examined visual word naming performance for high and low BOI words in a group of younger children (aged 6–7 years), a group of older children (aged 8–9 years), and a group of adults. To facilitate comparisons across the groups, Wellsby and Pexman used a composite measure of naming performance that combined naming latency and accuracy information. Results showed a significant facilitatory BOI effect for the older children and the adults but not for the younger children. For children, the size of their BOI effect was related to their age and also to their reading skills; that is, BOI effects were larger for older children and for children with higher scores on a standardized reading test. As such, Wellsby and Pexman concluded that the BOI variable begins to influence children’s lexical processing at about 8 years of age. In reasoning about why the effect does not emerge in younger children, Wellsby and Pexman concluded that two explanations were viable: first, younger children may not yet have had sufficient sensorimotor interactions with the words’ referents to create richer representations for high BOI items; second, younger children’s reading skills may not yet be efficient enough to support semantic feedback. Indeed, consistent with simulations of models of the lexical processing system (e.g., Plaut et al., 1996), it has been suggested that semantic factors become more important as children progress beyond the early stages of reading development (Nation, 2009; Hulme and Snowling, 2013).

There are, however, two issues with the methodology of the Wellsby and Pexman (2014a) study that may have limited detection of BOI effects in the younger children in that study. First, the use of the visual naming task may have limited the effects observed. Wellsby and Pexman reported that the visual naming task was very challenging for the younger children. The younger children made many more naming errors than the older children and also seemed to be sounding out the words, naming them in a step-by-step fashion, which could have complicated the measurement of naming latencies. Visual naming and lexical decision are traditional lexical tasks, yet these have rarely been used with children younger than 8 years of age. This is because in younger children, word reading skills are not advanced enough to support performance in visual naming or lexical decision. Further, in studies using these tasks, there is limited evidence for effects of sensorimotor variables like imageability and concreteness (Coltheart et al., 1988; McFalls et al., 1996). Since naming and lexical decision tasks require that children translate spelling into sound and meaning, performance in those tasks depends heavily on children’s reading skills and this could complicate the detection of sensorimotor effects.

A lingering question is thus: would children younger than 8 years of age show sensorimotor effects if given a lexical processing task that was less dependent on reading skills, particularly orthographic skills? To address this question, we chose an alternative task that has been used before to examine semantic effects in adults’ lexical processing (Tyler et al., 2000; Wurm et al., 2004). This is the auditory naming task, in which participants *hear* words and repeat each one aloud as quickly and as accurately as they can. Tyler et al. (2000) compared imageability effects in auditory naming and auditory lexical decision. They found imageability effects in both tasks, even when controlling for word length and familiarity. They concluded that even though the tasks have differences (auditory naming involves articulation while auditory lexical decision involves a decision component), both tasks “tap into the activation of semantic representations” (p. 324). Wurm et al. (2004) noted that unlike visual naming, processing stimuli in auditory naming does not require orthography to phonology translation. As such, the auditory naming task should depend less on orthographic coding skills than do tasks like visual naming and visual lexical decision but should still involve semantic processing. Younger children should be more proficient in an auditory naming task than in a visual naming task as their experience processing spoken language is far greater than their experience processing written language.

An additional issue with the previous examination of BOI effects in children’s lexical processing involves the use of adult BOI ratings. While many studies have used adult ratings of semantic variables to assess effects of those variables in children’s language processing, this practice is particularly problematic for BOI. The BOI variable is intended to capture fine-grained differences between highly imageable words; that is, it provides an indication of differences in the average person’s previous experiences physically interacting with the words’ referents. Whereas adults rate *axe* and *cane* as high BOI, these would likely not be objects with which children have extensive physical experience. While children may have plenty of visual experience with these objects, their physical experience will be different, due to their stature, permitted activities, and other constraints. As such, while adults’ ratings of imageability (which primarily assess visual information) are probably applicable to children, adults’ BOI ratings of their own bodily experience may not capture variability in children’s physical interaction experience. This could have been a particular issue for the younger children in the Wellsby and Pexman study (the 6–7 year olds), who would have had even less experience with some of the objects than the older children, and this issue could have contributed to the null BOI effect observed for that group. There is a need to ensure that the words chosen to assess BOI effects in children’s lexical processing are high and low in terms of the average *child’s* BOIs. This was addressed in the present study by asking parents of children in this age range (6–7 years) to provide ratings using a dimension we refer to as *child-BOI*. We reasoned that parents with children in this age group would be best suited to make ratings of 6–7 year olds’ sensorimotor experiences with word referents since these parents have direct knowledge of their children’s activities.

In the present study, we thus examined imageability effects (comparing responses to high and low imageability words) and child-BOI effects (comparing responses to high and low child-BOI words, which were a subset of the high imageability words) in the auditory naming task. We tested a group of 6–7 year old children and also a group of adults. Adults were tested primarily as a control group to verify our assumptions about the auditory naming task. That is, although the auditory naming task has been used to examine semantic effects in lexical processing with adults (i.e., Tyler et al., 2000; Wurm et al., 2004), we wanted to assess whether the task was sensitive to the semantic dimensions manipulated here. We included measures of children’s reading skills as an additional evaluation of the task. While children’s reading skills were correlated with aspects of their visual naming performance in Wellsby and Pexman (2014a), the auditory task used here should be less dependent on children’s orthographic skills. Thus we expected that reading skills would not correlated with task performance in the current study.

If BOI effects can be observed before age 8, but were masked in the previous study (Wellsby and Pexman, 2014a) by task difficulty or by items which did not account for adult-child differences in physical interaction experience, then we expected to find a child-BOI effect with 6–7 year olds using the present study’s items and auditory naming task. This would be evidence that sensorimotor information is part of children’s lexical knowledge, consistent with an embodied framework for word meaning. By this framework, we also expected imageability effects in the auditory naming task. It is also possible, however, that younger children may not have sufficient experience to afford richer semantic representations for high imageability and high child-BOI objects and thus we would not expect imageability and child-BOI effects to emerge before age 8.

## Materials and Methods

### Participants

Participants were 54 children aged 6–7 years (32 female, *M* age = 7;2, *SD* = 0;6) and 25 adults (14 female, *M* age = 21;2, *SD* = 2;4). Children were recruited through the University of Calgary Child and Infant Learning and Development (ChILD) database and received a small toy for participating. Adults were undergraduate students in Psychology courses at the University of Calgary who received partial course credit for participating.

### Stimuli

Stimuli for the auditory naming task consisted of 60 high imageability and 60 low imageability monosyllabic words. Imageability was determined based on published norms (Cortese and Fugett, 2004). To ensure that the words were all familiar to 6–7 year old children and that the high and low imageability word sets differed only in terms of imageability, the two word sets were not significantly different on all of the following characteristics: grade one print frequency (the frequency of a word in academic texts that tend to be part of the Grade 1 curriculum, as indexed by the Educator’s Word Frequency Guide, Zeno et al., 1995), children’s spoken frequency for the 6–7 year old age group in the Child Language Data Exchange System (CHILDES) database (from the ChildFreq lexical norms described in Bääth, 2010; which include frequency information for all words in CHILDES produced by an English child speaker, providing an estimate of the speech that children typically produce), adults’ spoken frequency in child-directed speech (the MacWhinney, 2000, CHILDES sub-corpus extracted by Ping Li, described here: http://childes.talkbank.org/derived/, providing an estimate of the speech that children are typically exposed to), word length, phonological Levenshtein distance (PLD; a measure of words’ phonological similarity, Yarkoni et al., 2008), orthographic Levenshtein distance (OLD; a measure of words’ orthographic similarity, Yarkoni et al., 2008), and valence (Warriner et al., 2013). Refer to **Table 1** for descriptive statistics of the stimuli.

**Table 1:** Mean characteristics of word stimuli (standard deviations in parentheses).

Word characteristic	High imageability	Low imageability	*p* for difference test	High child-BOI	Low child-BOI	*p* for difference test
Number of items	60	60		12	12	
Imageability	5.83 (0.78)	3.46 (0.60)	<0.001	6.40 (0.21)	6.34 (0.48)	0.38
Child-BOI				6.21 (0.59)	3.83 (1.36)	<0.001
Grade 1 print frequency	81.87 (58.30)	87.77 (70.62)	0.62	95.08 (64.84)	91.50 (42.75)	0.87
Child spoken frequency	87.02 (121.09)	81.50 (145.03)	0.82	164.08 (194.95)	65.58 (83.14)	0.13
Adult spoken frequency	291.72 (368.44)	245.93 (466.46)	0.55	604.42 (495.21)	304.25 (341.03)	0.10
Word length (letters)	4.28 (0.74)	4.30 (0.79)	0.91	4.17 (0.83)	4.58 (0.90)	0.25
PLD	1.29 (0.29)	1.23 (0.29)	0.27	1.23 (0.31)	1.31 (0.30)	0.53
OLD	1.46 (0.30)	1.47 (0.30)	0.90	1.46 (0.37)	1.58 (0.26)	0.35
Valence	5.23 (1.75)	5.30 (1.86)	0.83	6.46 (0.74)	5.65 (1.57)	0.12
Sound file length (ms)	382.67 (53.03)	395.65 (47.52)	0.16	376.92 (52.32)	384.50 (68.36)	0.76

*The high child-BOI low child-BOI words were a subset of the 60 high imageability words. BOI, body–object interaction; PLD, phonological Levenshtein distance; OLD, orthographic Levenshtein distance.*

To collect ratings of child-BOI, we presented a separate group of 16 adults (who were all parents to children in the same age range as child participants, but were not parents to the children who participated) with the 60 high imageability words. These adults were instructed to rate each word on a 7-point scale, and to use a 6-year-old child as their referent. That is, they were asked to rate how much physical interaction experience the average 6-year-old might be likely to have with each thing (1 = low, “things a typical child has not touched/held at all”; 7 = high, “things a typical child has touched/held a lot”). Based on these ratings, we selected 12 high child-BOI words (e.g., chair) and 12 low child-BOI words (e.g., knife), again not significantly different on grade one print frequency, child spoken frequency, adult spoken frequency, word length, PLD, OLD, and valence. Descriptive statistics for these items are also provided in **Table 1**. There was no significant correlation between child-BOI ratings and imageability ratings for the 60 high imageability words, *r* = 0.21, *p* = 0.10. This suggests that child-BOI and imageability are not strongly related constructs. The complete list of stimuli, including imageability and child-BOI ratings, can be found in the Appendix.

Word stimuli for the auditory naming task were recorded as sound files by a female speaker who was naïve to the purpose of the study (using Sound Recorder). Since all words were monosyllabic the durations of sound files were quite consistent; to further improve this, however, sound files were edited using the program Praat. The editing ensured that all files included the entire duration of the word and that there were no significant differences between lengths (durations) of the sound files for high imageability and low imageability word sets or for the high child-BOI and low child-BOI word sets (**Table 1**), while still maintaining perceptibility. Edited files were then played individually to six adults to verify that each word could still be recognized accurately.

### Procedure

Participants were tested in our university laboratory. They sat in front of a computer wearing headphones. A microphone was positioned in front of the child’s face, connected to a response box with voice key that measured the onset latency of vocal responses. The child either held the microphone or, if they preferred, the experimenter did so. The experimenter sat beside the participant, and also wore headphones. Sound files were presented one at a time through both the participant’s and the experimenter’s headphones using E-Prime presentation software (Schneider et al., 2001). Participants were instructed to repeat each word into the microphone immediately after hearing it, and to do so loudly and clearly. The task began with five practice trials, followed by a short break for participants to ask any questions, and then the 120 experimental trials. The words were presented in randomized order to each participant. During the practice and experimental trials, the screen was blank except for a small fixation cross in the center.

Once a word was vocalized, the researcher coded the response as “correct” (participant repeated the correct word), “incorrect” (participant did not repeat the correct word), or “spoiled” (participant hesitated, stuttered, or prematurely triggered the response box) using the computer keyboard. The code of 1, 2, or 3 (respectively) prompted the presentation of the next word; this way, the experiment proceeded at a pace the participant was comfortable with. Participants were also allowed to request a break at any point in order to prevent fatigue effects.

Following the auditory naming task, child participants completed three subtests of the Woodcock Reading Mastery Tests-Revised (WRMT-R; Woodcock, 1997). These represent a subset of the tests administered in the Wellsby and Pexman (2014a) study. These subtests were administered to obtain a measure of participants’ reading ability across three different dimensions: their letter identification skills (i.e., what letter is this?), word reading skills (i.e., what word is this?), and orthographic-phonological conversion skills (“word attack,” i.e., how does this non-word sound?). Children’s mean scores on these subtests (letter identification *M* = 38.58, *SD* = 3.57, word reading *M* = 47.50, *SD* = 21.89 and word attack *M* = 19.56, *SD* = 10.20) were very similar to those obtained for the younger group in the Wellsby and Pexman study.

## Results

We first examined auditory naming accuracy by item to determine whether any items should be excluded from the analysis. The item “full” was removed as it was only named correctly 24.32% of the time by the child participants. Removing this low imageability item did not compromise the matching illustrated in **Table 1**. Auditory naming accuracy was high for all other items (97.17% for children, 98.69% for adults): too high, in fact, to warrant further analysis of the accuracy data. Before analyzing latency data, we removed latencies for trials with incorrect (2.83% for children, 1.18% for adults) or spoiled responses (0.81% for children, 0.13% for adults). In addition, trials with response latencies faster than 250 ms or slower than the participant’s mean plus 2.5 SD (3.92% for children, 1.38% for adults) were removed from the analysis. Since the adults were tested simply to establish that the auditory naming task was sensitive to the kinds of effects tested here, we present the analysis of the adult data first and then, separately, analysis of the child data, including tests for correlations with reading skill variables. Imageability effects in response latencies were examined using ANOVAs on the entire dataset [119 items, with imageability (high vs. low) as a fixed factor], whereas child-BOI effects were examined using separate ANOVAs on the subset of the high imageability items for which child-BOI was manipulated [24 items, with child-BOI (high vs. low) as a fixed factor].

### Adults

The analysis of adults’ auditory naming latencies showed a significant effect of imageability, *F*(1,24) = 28.46, *p* < 0.001, $\eta_{p}^{2}=0.543$. That is, adults named high imageability words (*M* = 752.70, *SD* = 77.37) faster than low imageability words (*M* = 768.32, *SD* = 76.27). Although it was not our focus for the adult data, we also tested the effect of child-BOI for adults and found that it was not significant, *F*(1,24) = 3.99, *p* = 0.057, $\eta_{p}^{2}=0.142$. Adults did not name high child-BOI words (*M* = 746.81, *SD* = 76.33) significantly faster than low child-BOI words (*M* = 755.43, *SD* = 81.91). Given that child-BOI was characterized based on 6-year-olds’ physical experience this is not entirely surprising.

### Children

The analysis of children’s auditory naming latencies showed a significant effect of imageability, *F*(1,53) = 57.02, *p* < 0.001, $\eta_{p}^{2}=0.518$. That is, children named high imageability words (*M* = 881.96, *SD* = 123.02) faster than low imageability words (*M* = 906.91, *SD* = 122.12). There was also a significant effect of child-BOI for children’s naming latencies, *F*(1,53) = 44.86, *p* < 0.001, $\eta_{p}^{2}=0.458$: Children named high child-BOI words (*M* = 860.01, *SD* = 126.19) faster than low child-BOI words (*M* = 899.25, *SD* = 132.36).

We next examined correlations between children’s age, reading skills, imageability effects, and child-BOI effects. These correlations are presented in **Table 2**. Not surprisingly, there were significant correlations between children’s age and scores on each of the reading subtests, since older children also tended to have better reading skills. In addition, children’s scores on the reading subtests were correlated. More importantly, children’s age was correlated with their child-BOI effect but not their imageability effect, such that older children showed larger BOI effects. There were no significant relationships between the reading skill measures and the imageability or child-BOI effects, and this held regardless of whether age was partialled out of the correlations. There was also no significant relationship between the size of children’s imageability and child-BOI effects.

**Table 2:** Correlations among variables.

Variable	2	3	4	5	6
(1) Child age in months	0.01	0.40ˆ**	-0.56ˆ***	0.54ˆ***	0.49ˆ***
(2) Imageability effect	–	0.10	0.01	–0.09	–0.03
(3) Child-BOI effect	–0.12	–	0.24	0.23	0.12
(4) WRMT: letter identification	0.00	0.03	–	0.71ˆ***	0.69ˆ***
(5) WRMT: word reading	–0.12	0.02	0.59ˆ***	–	0.91ˆ***
(6) WRMT: word attack	–0.05	–0.10	0.58ˆ***	0.88ˆ***	–

*Raw correlations are presented above the diagonal, and partial correlations (controlling for age) are presented below the diagonal. BOI, body–object interaction; WRMT, Woodcock Reading Mastery Test. ^∗∗^p < 0.01, ^∗∗∗^p < 0.001.*

In collecting ratings of the child-BOI dimension in the present study, we asked parents to rate “how much experience the average 6-year-old would have likely had physically interacting (using the body: hands, mouth, etc)” with each word’s referent. In this way our ratings instructions were somewhat different than those used by Siakaluk and colleagues to collect BOI ratings from adults (e.g., Tillotson et al., 2008). The BOI ratings instructions for adults emphasized *ease* of interaction. Here, for child-BOI ratings, we emphasized *likelihood* of interaction in order to highlight the ways in which a child’s experience might be different than that of adults. As such, the child-BOI dimension probably measures a slightly different aspect of interaction than does the adult BOI dimension, and frequency of interaction may play a stronger role in child-BOI ratings than in adult-BOI ratings. Further, while the low and high child-BOI word sets do not differ significantly on print frequency or on either of the spoken frequency measures (child spoken frequency, adult spoken frequency), there are large numeric differences between the low and high child-BOI word sets on the spoken frequency dimensions. To evaluate whether the child-BOI effect we observed was incremental to those spoken frequency differences, we conducted linear mixed effects analyses (Baayen et al., 2008) using R. The influence of spoken frequency (either child or adult, in separate models), age, and child-BOI rating were treated as fixed effects, while participants and items were treated as random variables. The dependent measure was auditory naming latency. We began with models including spoken frequency, child-BOI rating and child’s age as predictors. However, since child-BOI effects tended to be larger for older children, we also tested the inclusion of an interaction of child-BOI rating and child’s age as a predictor. We compared the models with the interaction term to models without the interaction term, and found that including the interaction as a predictor significantly improved model fit: χ^2^(1) = 4.43, *p* = 0.035 for the model in **Table 3**, with child spoken frequency as a predictor, and χ^2^(1) = 4.45, *p* = 0.035 for the model in **Table 4**, with adult spoken frequency as a predictor. The resulting model with child spoken frequency as a predictor is presented in **Table 3**, and the model with adult spoken frequency as a predictor is presented in **Table 4**. As can be seen, even after including spoken frequency as a predictor, there is evidence that effects of child-BOI may be incremental to the influence of spoken frequency.

**Table 3:** Summary of regression model predicting child reaction time, with child spoken frequency as a predictor.

Fixed Effect	Coefficient	*SE*	*t*	*p*
Intercept	–286.94	273.57	–1.05	0.30
Child spoken frequency	–0.13	0.07	–1.78	0.09
Child-BOI rating	60.93	33.60	1.81	0.07
Age	14.26	3.16	4.52	<0.001ˆ***
Child-BOI rating × Age	–0.81	0.38	–2.11	0.04ˆ*

**Random effect**	***s*^2^**			

Subject intercept	11423.33			
Item intercept	1886.17			

*N = 1184; log-likelihood = 7472.08; AIC = 14960.16. ^∗^p < 0.05, ^∗∗∗^p <0.001.*

**Table 4:** Summary of regression model predicting child reaction time, with adult spoken frequency as a predictor.

Fixed effect	Coefficient	*SE*	*t*	*p*
Intercept	289.47	273.71	1.06	0.29
Adult spoken frequency	0.04	0.03	1.56	0.13
Child-BOI rating	62.60	33.79	1.85	0.06
Age	14.26	3.16	4.52	<0.001ˆ***
Child-BOI rating × Age	0.81	0.38	2.11	0.04ˆ*

**Random effect**	***s*^2^**			

Subject intercept	11423.33			
Item intercept	1950.11			

*N = 1184; log-likelihood = 7472.42; AIC = 14960.84. ^∗^p < 0.05, ^∗∗∗^p < 0.001.*

## Discussion

The purpose of the present research was to examine whether children’s lexical processing is influenced by words’ sensorimotor histories (as indexed by imageability and child-BOI) before age 8. Children younger than 8 years of age typically struggle with lexical processing tasks that depend on reading skills. With this in mind, we used an auditory naming task to assess the influence of sensorimotor variables in 6- and 7-year-old children. Even the youngest children in the sample were able to perform this task with high accuracy, suggesting it was well suited to their lexical processing skills. Results showed a significant imageability effect and a significant child-BOI effect in this age group. Thus, there is now evidence that children’s lexical processing is influenced by sensorimotor variables before 8 years of age.

Our findings suggest that children do have richer semantic representations for words associated with more sensorimotor information. Thus, sensorimotor information is part of children’s lexical knowledge, consistent with an embodied framework for word meaning. Although these representations may not be measurable in orthographically based tasks until age 8, effects can be observed earlier in a word processing task that does not require orthographic coding. As such, our results provide an important new data point for sensorimotor effects in childhood and are consistent with the assertion that word meaning is embodied throughout the lifespan. This conclusion would be compatible with results showing that during passive listening to verb stimuli (e.g., chase, clap) 4- to 6-year-old children showed activation in the motor cortex (James and Maouene, 2009). Similarly, Maouene et al. (2008) found that the earliest acquired verbs tended to be those associated with the mouth, with verbs associated with hand and arm actions acquired next, and that verbs not associated with any particular body part were acquired later. That is, in the 2nd and 3rd years of life verbs enter children’s productive vocabularies at a rapid rate, and the order of acquisition can be predicted, to some degree, by the relationship of the verb to the child’s developing motor system.

The present results also showed that while the child-BOI effect in auditory naming was related to age, the imageability effect was not. That is, child-BOI effects tended to increase across the developmental window examined, while imageability effects remained stable. The child-BOI variable is intended to measure physical experiences, and these may be accumulating rapidly in this age group, such that the older children in the sample have sufficiently more physical interaction experiences to effect differences in the underlying semantic representations. In contrast, imageability measures multisensory (visual, sound, etc.) experiences. Since these multisensory experiences do not depend on motor skills to the same degree that child-BOI-related experiences do, they may begin to accumulate earlier and reach a relative plateau during the developmental window assessed here. In future studies, it would be important to test an even younger group of children to determine when imageability effects are first observed in children’s lexical processing. Given the high level of accuracy children exhibited for the auditory naming task in the present study, it may be possible to use this task successfully with an even younger group of children in order to test these remaining research questions.

Additional topics for future research include investigating implications for vocabulary acquisition and literacy development: if bodily experiences are an important aspect of children’s semantic representations, then providing more of those experiences may facilitate more rapid acquisition of word meaning. In related research, James and Swain (2011) used fMRI to explore differences in the neural correlates of verbs learned through self-generated actions vs. observed actions. Children aged 5–6 years were taught novel verb labels while performing an action on an object or watching an experimenter perform an action on an object. Later, during an fMRI session, children heard the verbs and saw photographs of the objects but did not make a behavioral response. Results showed activity in the motor cortex during passive listening to verbs that had been learned through self-generated actions but not for verbs learned through observed actions. Similarly, children’s motor systems were more active when viewing objects experienced through active interaction than when viewing objects experienced through observed interactions. These findings suggest that children’s representations of verb meaning involve more motor information if those verbs are learned through direct physical experience with the actions implied. James and Swain (2011) did not test whether these representational differences had behavioral consequences, so we don’t yet know whether physical experiences facilitate acquisition of word meaning, but this will be an important issue to address in order to more fully understand the applications of this research.

The present results provide new insight about the developing lexicon and suggest a role for sensorimotor experience in the acquisition of word meaning. Sensorimotor effects can be observed in auditory naming in 6–7 year olds, but future research will be required to map out the trajectory of sensorimotor effects in the developing linguistic and conceptual systems.

## Author Contributions

MI collected most of the data and wrote the first draft of the manuscript. MW helped design and program the experiment and assisted with analyses. EL assisted with data collection, coding, and writing. PP helped design the experiment, analyze the data, and write the paper.

## Conflict of Interest Statement

The authors declare that the research was conducted in the absence of any commercial or financial relationships that could be construed as a potential conflict of interest.

## References

[B1] Bääth R. (2010). ChildFreq: an online tool to explore word frequencies in child language. *LUCS Minor* 16 1–6.

[B2] Baayen R. H. Davidson D. J. Bates D. M. (2008). Mixed-effects modeling with crossed random effects for subjects and items. *J. Mem. Lang.* 59 390–412. 10.1016/j.jml.2007.12.005

[B3] Coltheart V. Laxon V. J. Keating C. (1988). Effects of word imageability and age of acquisition on children’s reading. *Br. J. Psychol.* 79 1–12. 10.1111/j.2044-8295.1988.tb02270.x

[B4] Cortese M. J. Fugett A. (2004). Imageability ratings for 3,000 monosyllabic words. *Behav. Res. Methods Instr. Comput.* 36 384–387. 10.3758/BF03195585 15641427

[B5] Cortese M. J. Simpson G. B. Woolsey S. (1997). Effects of association and imageability on phonological mapping. *Psychon. Bull. Rev.* 4 226–231. 10.3758/BF03209397 21331829

[B6] Glenberg A. (2015). Few believe the world is flat: how embodiment is changing the scientific understanding of cognition. *Can. J. Exp. Psychol.* 69 157–163. 10.1037/cep0000056 26010024

[B7] Harm M. W. Seidenberg M. S. (2004). Computing the meanings of words in reading: cooperative division of labor between visual and phonological processes. *Psychol. Rev.* 111 662–720. 10.1037/0033-295X.111.3.662 15250780

[B8] Hino Y. Lupker S. J. (1996). Effects of polysemy in lexical decision and naming: an alternative to lexical access accounts. *J. Exp. Psychol.* 22 1331–1356.

[B9] Hulme C. Snowling M. J. (2013). Learning to read: what we know and what we need to understand better. *Child Dev. Perspect.* 7 1–5. 10.1111/cdep.12005 26290678 PMC4538787

[B10] James K. H. Maouene J. (2009). Auditory verb perception recruits motor systems in the developing brain: an fMRI investigation. *Dev. Sci.* 12 F26–F34. 10.1111/j.1467-7687.2009.00919.x 19840036

[B11] James K. H. Swain S. N. (2011). Only self-generated actions create sensori-motor systems in the developing brain. *Dev. Sci.* 14 673–678. 10.1111/j.1467-7687.2010.01011.x 21676088 PMC4176697

[B12] MacWhinney B. (2000). *The Childes Project*, 3rd Edn. Mahwah, NJ: Erlbaum.

[B13] Maouene J. Hidaka S. Smith L. B. (2008). Body parts and early-learned words. *Cogn. Sci.* 32 1200–1216. 10.1080/03640210802019997 21585449

[B14] McFalls E. L. Schwanenflugel P. J. Stahl S. A. (1996). Influence of word meaning on the acquisition of a reading vocabulary in second-grade children. *Read. Writ.* 8 235–250. 10.1007/BF00420277

[B15] Meteyard L. Rodriguez Cuadrado S. Bahrami B. Vigliocco G. (2012). Coming of age: a review of embodiment and the neuroscience of semantics. *Cortex* 48 788–804. 10.1016/j.cortex.2010.11.002 21163473

[B16] Nation K. (2009). Form-meaning links in the development of visual word recognition. *Philos. Trans. R. Soc. B* 364 3665–3674. 10.1098/rstb.2009.0119 PMC2846312 19933139

[B17] Plaut D. C. McClelland J. L. Seidenberg M. S. Patterson K. (1996). Understanding normal and impaired word reading: computational principles in quasi-regular domains. *Psychol. Rev.* 103 56–115. 10.1037/0033-295X.103.1.56 8650300

[B18] Schneider W. Eschman A. Zuccolotto A. (2001). *E-Prime user’s Guide*. Pittsburgh, PA: Psychology Software Tools.

[B19] Siakaluk P. D. Pexman P. M. Aguilera L. Owen W. J. Sears C. R. (2008a). Evidence for the activation of sensorimotor information during visual word recognition: the body-object interaction effect. *Cognition* 106 433–443. 10.1016/j.cognition.2006.12.011 17258186

[B20] Siakaluk P. D. Pexman P. M. Sears C. R. Wilson K. Locheed K. Owen W. J. (2008b). The benefits of sensorimotor knowledge: body-object interaction facilitates semantic processing. *Cogn. Sci.* 32 591–605. 10.1080/03640210802035399 21635348

[B21] Strain E. Patterson K. Seidenberg M. S. (1995). Semantic effects in single-word naming. *J. Exp. Psychol.* 21 1140–1154. 10.1037//0278-7393.21.5.1140 8744959

[B22] Tillotson S. M. Siakaluk P. D. Pexman P. M. (2008). Body-Object interaction ratings for 1,618 monosyllabic nouns. *Behav. Res. Methods* 40 1075–1078. 10.3758/BRM.40.4.1075 19001398

[B23] Tyler L. K. Voice K. Moss H. E. (2000). The interaction of meaning and sound in spoken word recognition. *Psychon. Bull. Rev.* 7 320–326. 10.3758/BF03212988 10909140

[B24] Warriner A. B. Kuperman V. Brysbaert M. (2013). Norms of valence, arousal, and dominance for 13,915 English lemmas. *Behav. Res. Methods* 45 1191–1207. 10.3758/s13428-012-0314-x 23404613

[B25] Wellsby M. Pexman P. M. (2014a). The influence of bodily experience on children’s language processing. *Top. Cogn. Sci.* 6 425–441. 10.1111/tops.12092 25060918

[B26] Wellsby M. Pexman P. M. (2014b). Developing embodied cognition: insights from children’s concepts and language processing. *Front. Psychol.* 5:506 10.3389/fpsyg.2014.00506 PMC4036138 24904513

[B27] Woodcock R. N. (1997). *Woodcock Reading Mastery Tests-Revised/Normative Update*. Circle Pines, MN: American Guidance Service.

[B28] Wurm L. H. Vakoch D. A. Seaman S. R. (2004). Recognition of spoken words: semantic effects in lexical access. *Lang. Speech* 47 175–204. 10.1177/00238309040470020401 15581191

[B29] Yarkoni T. Balota D. Yap M. (2008). Moving beyond Coltheart’s N: a new measure of orthographic similarity. *Psychon. Bull. Rev.* 15 971–979. 10.3758/PBR.15.5.971 18926991

[B30] Zeno S. M. Ivens S. H. Millard R. T. Duvvuri R. (1995). *The Educator’s Word Frequency Guide*. Brewster, NY: Touchstone Applied Science Associates Inc.

