# Supplementary material for: Development of Embodied Word Meanings: Sensorimotor Effects in Children’s Lexical Processing
Source: Front Psychol. 2016 Mar 8;7:317. doi: 10.3389/fpsyg.2016.00317 (PMC4782215; doi:10.3389/fpsyg.2016.00317)
Supplement: Supplementary file 1 [file Data_Sheet_1.DOCX]

Appendix

Words presented in the auditory naming task,

with imageability ratings and (where relevant) child-BOI ratings

| High Imageability Words | | | Low Imageability Words | |
| --- | --- | --- | --- | --- |
| Word | Imageability | Child-BOI | Word | Imageability |
| bag^H^ | 6.4 | 6.19 | bright | 4.4 |
| cake^H^ | 6.8 | 6.56 | build | 3.8 |
| chair^H^ | 6.5 | 6.75 | calm | 2.8 |
| child^H^ | 6.4 | 6.31 | chance | 2.1 |
| doll^H^ | 6.4 | 5.63 | change | 3.2 |
| fruit^H^ | 6.3 | 6.63 | cold | 4.2 |
| hair^H^ | 6.3 | 6.88 | dare | 2.5 |
| juice^H^ | 6.3 | 6.56 | death | 4.0 |
| kite^H^ | 6.5 | 5.75 | dumb | 3.5 |
| map^H^ | 5.9 | 5.31 | fear | 2.9 |
| pet^H^ | 6.4 | 5.19 | fierce | 3.6 |
| phone^H^ | 6.6 | 6.75 | fool | 3.7 |
| beach^L^ | 6.5 | 5.69 | free | 3.0 |
| blood^L^ | 6.4 | 3.06 | fresh | 3.2 |
| gun^L^ | 6.6 | 2.19 | full | 3.4 |
| heart^L^ | 6.7 | 3.06 | glad | 3.0 |
| knife^L^ | 6.8 | 3.69 | grand | 2.7 |
| nest^L^ | 6.2 | 3.38 | hit | 4.4 |
| prince^L^ | 6.4 | 2.44 | hold | 4.0 |
| smoke^L^ | 6.4 | 2.75 | hope | 3.0 |
| tea^L^ | 6.4 | 3.13 | joke | 3.6 |
| train^L^ | 6.3 | 5.75 | joy | 3.7 |
| game^L^ | 4.9 | 5.94 | late | 3.7 |
| smile^L^ | 6.5 | 4.94 | law | 3.6 |
| cheer | 4.6 | 3.88 | lie | 3.2 |
| clean | 4.5 | 4.50 | life | 3.9 |
| cry | 5.3 | 4.94 | lose | 3.0 |
| cut | 5.1 | 3.00 | love | 4.0 |
| dance | 5.6 | 4.94 | luck | 2.4 |
| dead | 5.3 | 1.81 | mad | 3.8 |
| draw | 5.0 | 5.56 | mess | 4.2 |
| fall | 4.8 | 3.50 | neat | 3.5 |
| fight | 4.8 | 3.88 | nice | 3.0 |
| hunt | 4.6 | 2.31 | noise | 3.9 |
| jump | 4.8 | 5.31 | noon | 4.0 |
| kill | 4.8 | 1.81 | pain | 4.3 |
| lead | 4.7 | 2.69 | plain | 3.5 |
| nap | 4.7 | 4.25 | poor | 4.0 |
| ride | 4.6 | 4.63 | proud | 2.9 |
| treat | 4.6 | 5.94 | prove | 2.4 |
| type | 5.4 | 3.31 | quick | 2.9 |
| team | 5.0 | 3.56 | quit | 2.9 |
| jail | 6.6 | 2.06 | raise | 4.0 |
| rose | 6.7 | 4.25 | raw | 4.3 |
| cage | 6.4 | 3.69 | reach | 4.3 |
| cave | 6.7 | 3.75 | rise | 3.3 |
| cliff | 6.3 | 2.25 | rough | 3.2 |
| dust | 5.2 | 3.81 | sad | 3.9 |
| junk | 5.4 | 3.88 | save | 2.9 |
| lake | 6.6 | 5.19 | sense | 2.8 |
| mate | 4.8 | 2.75 | slow | 3.4 |
| park | 6.5 | 6.00 | smell | 3.4 |
| rat | 6.8 | 2.44 | sweet | 4.0 |
| skunk | 6.5 | 2.56 | trust | 2.9 |
| slave | 5.7 | 1.31 | waste | 3.8 |
| sweat | 5.8 | 4.63 | wild | 4.1 |
| tramp | 4.9 | 1.81 | win | 4.0 |
| trash | 6.2 | 4.44 | wise | 2.9 |
| wheat | 6.5 | 3.88 | wrong | 2.4 |
| whip | 5.0 | 2.31 | trap | 4.4 |

*Note*. Words with superscript H comprised the 12 high child-BOI items; words with superscript L comprised the 12 low child-BOI items. Imageability values are from Cortese & Fugett (2004). Child-BOI values are mean ratings from the present study.
